# Cultural adaptation is maximised when intelligent individuals rarely think for themselves

**DOI:** 10.1017/ehs.2020.42

**Published:** 2020-08-10

**Authors:** Elena Miu, Thomas J. H. Morgan

**Affiliations:** School of Human Evolution and Social Change and Institute of Human Origins, Arizona State University, Tempe, AZ 85287, USA

**Keywords:** cultural evolution, social learning, learning mechanisms, recombination

## Abstract

Humans are remarkable in their reliance on cultural inheritance, and the ecological success this has produced. Nonetheless, we lack a thorough understanding of how the cognitive underpinnings of cultural transmission affect cultural adaptation across diverse tasks. Here, we use an agent-based simulation to investigate how different learning mechanisms (both social and asocial) interact with task structure to affect cultural adaptation. Specifically, we compared learning through refinement, recombination or both, in tasks of different difficulty, with learners of different asocial intelligence. We find that for simple tasks all learning mechanisms are roughly equivalent. However, for hard tasks, performance was maximised when populations consisted of highly intelligent individuals who nonetheless rarely innovated and instead recombined existing information. Our results thus show that cumulative cultural adaptation relies on the combination of individual intelligence and ‘blind’ population-level processes, although the former may be rarely used. The counterintuitive requirement that individuals be highly intelligent, but rarely use this intelligence, may help resolve the debate over the role of individual intelligence in cultural adaptation.

## Graphical abstract

**Figure:**
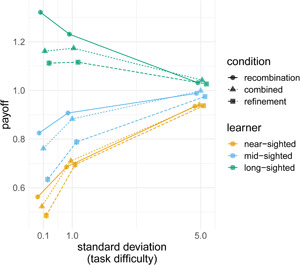

**Media summary**: Agents learning through recombination perform better than through refinement, particularly for difficult tasks.

## 1. Introduction

Cumulative cultural evolution – the process through which we build upon knowledge inherited over time (henceforth CCE) – has allowed our species to achieve the astounding ecological success witnessed today. Multiple factors, from psychology to demography, are argued to be integral to this process, including cooperation, high-fidelity social learning (Dean, Kendal, Schapiro, Thierry, & Laland, 2012; Richerson & Boyd, 2005; Tomasello, 1999) and population size and connectedness (Derex, Beugin, Godelle, & Raymond, 2013; Henrich, 2004; Kline & Boyd, 2010). However, the forces that shape CCE remain unclear, as do the reasons why it is effectively unique to our species.

Theoretical approaches have considered the conditions under which CCE is adaptive (Boyd & Richerson, 1996), the importance of faithful knowledge transmission (Enquist & Ghirlanda, 2007; Enquist, Strimling, Eriksson, Laland, & Sjostrand, 2010; Lewis & Laland, 2012) and the role of demography (Henrich, 2004). However, despite the fact that CCE often produces traits beyond the ability of any single individual, the modelling literature has typically operationalised CCE in simple ways, such as the persistence of traits in a population (Enquist & Ghirlanda, 2007; Enquist et al., 2010), the total number of traits (Enquist, Ghirlanda, Jarrick, & Wachtmeister, 2008; Lehmann, Aoki, & Feldman, 2011; Lehmann, Feldman, & Kaeuffer, 2010; Strimling, Sjöstrand, Enquist, & Eriksson, 2009) or the continuous/sequential improvement of a single trait (Henrich, 2004; Mesoudi, 2011; Morgan, 2016).

This way of modelling CCE as linear progression towards complexity is useful and intuitive, but a far cry from human culture where traits are often highly complex, consisting of multiple parts that interact in non-obvious ways. To model the effect of this complexity on CCE, Enquist et al. (2011) used a framework including dependencies between cultural traits, ranging from sequential improvement to differentiation, recombination and entire systems of interacting traits. They found that different dependency frameworks resulted in different rates of growth in the cultural traits and different diversity patterns. Thus, the way we model the structure of the cultural environment has important implications for our understanding of cultural evolution.

Empirical work points in the same direction. Laboratory studies using transmission chains have found evidence of improvement over time in a variety of tasks like building paper airplanes, spaghetti towers (Caldwell & Millen, 2008, 2010), virtual fishing nets (Derex, Godelle, & Raymond, 2013) or virtual arrowheads (Derex, Beugin, et al., 2013). Most of this work has tested the hypothesis that larger groups maintain more and more complex culture, some finding evidence for it (Derex, Beugin, et al., 2013; Muthukrishna, Shulman, Vasilescu, & Henrich, 2013), while some finding none (Caldwell & Millen, 2010; Fay, De Kleine, Walker, & Caldwell, 2019). The reason behind the conflicting results remains an active debate (Andersson & Read, 2016; Vaesen, Collard, Cosgrove, & Roebroeks, 2016), but one possibility is that some of the tasks used may not be complex enough to model human culture: if the task is easy enough to be solved individually, then larger groups would not add benefits. Therefore, results from both the theoretical and empirical literature emphasise the need to investigate CCE using appropriately complex tasks and cultural systems.

A broader issue is the lack of a clear mechanistic understanding of CCE. In Darwinian terms, CCE requires variation in, inheritance of and selection between cultural traits. Selection has been examined in the form of social learning biases (Enquist, Eriksson, & Ghirlanda, 2007; Henrich & Gil-White, 2001; Morgan, Rendell, Ehn, Hoppitt, & Laland, 2012). Inheritance has also been considered, although largely in terms of the fidelity of transmission (Fogarty, Strimling, & Laland, 2011; Lewis & Laland, 2012). However, little attention has been given to the sources of variation in cultural traits (although see Fogarty, Creanza, & Feldman, 2015 for a review). The most commonly cited sources are pure serendipity and refinement, whereby individuals improve inherited traits through small modifications (Basalla, 1988; Caldwell & Millen, 2008; Miu, Gulley, Laland, & Rendell, 2018). Other variation-generating mechanisms have been neglected. Recombination (i.e. combining cultural traits that already exist in the population), for instance, is cited as a key driver of CCE, but evidence is scarce. In a transmission chain study in which participants reproduced graphical symbols using complex software, Muthukrishna et al. (2013) observed that, when presented with several models, participants combined features from sources rather than copying just one. Similarly, Derex et al. (2015) found that participants combined and transformed information from multiple sources to produce new solutions, and a study of innovation in US patents also finds that combination is a key process in innovation (Youn, Strumsky, Bettencourt, & Lobo, 2015). Indeed, Muthukrishna and Henrich (2016) argue that recombination is as important as serendipity and refinement, and suggest that each will lead to different population level outcomes.

With this in mind, a recent experimental study examines CCE in a context where recombination is essential: the landscape of possible traits was structured as a branching tree, where high-payoff traits required the combination of traits from multiple branches (Derex, Perreault, & Boyd, 2018). Participants were arranged in groups with different levels of intra-connectedness, and partially connected populations (as opposed to fully connected populations) discovered more successful traits. This reflects a trade-off between exploring new traits and exploiting known traits: fully connected groups quickly converged on similar traits, while partially connected groups maintained more diversity which, in turn, fuelled further discoveries through recombination. This experiment is able to provide insight into the relationship between population size and cultural complexity, precisely because it uses a novel task structure and source of variation (Acerbi, Tennie, & Mesoudi, 2016; Aoki, Wakano, & Lehmann, 2012; Henrich, 2004; Mesoudi, 2011; Nakahashi, 2014). Other studies support the conclusion that both too little and too much connectivity hinders performance (Derex et al., 2018; Mason, Jones, & Goldstone, 2008): fragmented populations find better solutions for problems that benefit from broad exploration, while fully connected populations efficiently solve easier problems (Lazer & Friedman, 2007). Thus, task difficulty and task structure moderate the effect of demography on CCE.

Taken together, these results suggest that cumulative cultural evolution depends on complex interactions between the task structure and the mechanisms of inheritance and innovation. Here, we present a systematic investigation of this interaction. We used an agent-based model that aims to be a suitably complex representation of the remarkably complex solutions that cumulative cultural evolution produces in response to very difficult problems. One such example is cassava or manioc processing. Cassava root is one of the main sources of starch consumed in the tropics, but the root contains cyanide and without proper processing it can lead to goiter (swollen neck), neurological problems, paralysis and death. The proper processing of cassava consists of multiple steps including fermentation, drying and cooking over a period of several days (Henrich, 2016). Deviating from this process is risky, as the costs of errors are very high. Moreover, the chemical process is opaque, meaning that it is hard to know which part of the process caused any detrimental side effects, particularly since some of the symptoms develop only after repeated consumption of ill-processed cassava. Given this, the cultural evolution of safe cassava processing is all the more remarkable. Nonetheless, there are numerous such examples of multistep solutions to extremely difficult problems in human history, for example: nixtamalisation – soaking maize in an alkaline solution in order to increase nutritional value and reduce toxins (Staller & Carrasco, 2010); making cordage (Clarke, 2012); and Inuit clothing (Issenman, 2011).

Therefore here, using a novel task, inspired by the functional domain of tool-making, that involves dependencies between subunits, we explore how task difficulty (the extent of the dependencies) interacts with the mechanisms of inheritance and innovation (refinement and recombination) to affect cumulative cultural evolution. We find that the benefit of each inheritance mechanism is modulated by task difficulty, with recombination, unexpectedly, outperforming refinement on hard tasks even when individuals are skilled refiners.

## 2. Methods

We simulated a population of *n_a_* agents who each learn a repertoire consisting of 10 fitness-relevant steps. For each step agents choose one of 10 options, and the option selected can modify the fitness effect of options chosen for other steps. As such, an agent's repertoire can be conceptualised as a series of separate-but-interconnected steps, or a single multi-step process.

It is worth noting that although multi-step processes such as manioc processing are handy illustrations for the type of processes we had in mind, our model is more general. The steps can be conceptualised as sequentially dependant elements forming an overall process, but such a sequential interpretation is not necessary. The steps can be more generally conceptualised as elements that depend on each other, but do not have to be performed in a specific order. A step being fourth in the repertoire does not mean that it is performed fourth in some sequence, rather it just means that its payoff depends on steps 2 and 3. As such, agents can learn and modify steps in any order.

Repertoires are shared between agents via intergenerational cultural transmission across non-overlapping generations. The initial generation of agents acquires its repertoire through asocial learning alone, while subsequent generations also have access to social information from the most recent prior generation. This process repeats for a total of 60 generations (we explored different values for the number of generations and found little difference in the main results; see Figure S1 – we chose 60 generations for the main paper and 30 generations for the Supplementary Material). As described below, across simulations we varied task difficulty, agent asocial intelligence, and the mechanism of cultural inheritance.

### 2.1. The task

Agents learn a culturally heritable repertoire of 10 steps. For each step, there are 10 mutually exclusive options. We will use *o_s_* to denote the option chosen for step *s* (i.e. *o*_2_ = 5 indicates that option 5 was selected for step 2). Each option for each step is associated with its own payoff, yet agents will not always receive the full value of this payoff owing to interactions between options across different steps, which penalise payoffs. Therefore, we distinguish between an option's ‘base’ payoff and an agent's ‘received’ payoff from that option. An agent's total payoff is the sum of the received payoffs from the options they choose.

**Figure 1.**
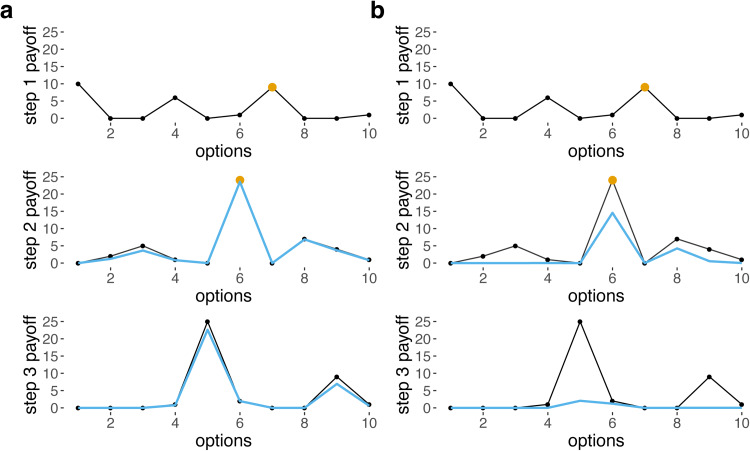
Base payoffs (black) and received payoffs given choices made at previous steps (blue) for 10 options for steps 1–3, when (a) *σ* = 5, ‘easy’ and (b) *σ* = 1, ‘moderate’. Yellow dots indicate the option chosen for each step. Received payoffs for step 2 in blue are calculated assuming that option 7 was chosen for step 1, and received payoffs for step 3 are calculated assuming that option 7 was chosen for step 1 and option 6 was chosen for step 2. Note that there is no blue line shown for step 1 because, for the first step, base payoffs are identical to received payoffs. When *σ* is lower and the task is harder, the penalties are much harsher (b).

The *base* payoff for each option *p*(*s, o_s_*) chosen for step *s* is randomised for each simulation and is drawn from an exponential distribution with a rate of 1, then squared, doubled and rounded. This produces a distribution of payoffs (i.e. payoff matrix) with a few high payoff options for each step, and many low or zero payoff options (Figs 1 and S2), because the types of problem we are modelling are characterised by sparse fitness landscapes with very few high peaks, where the costs of error are high.

The *received* payoff from option *o_s_*, *ρ*(*s*, *o_s_*) is calculated by multiplying the option's base payoff, *p*(*s*, *o_s_*), by the values of a Gaussian function (centred on *o_s_*, and denoted *G*) at points *o_s_*_−1_ and *o_s_*_−2_:1
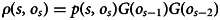
where:2
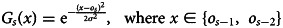


The Gaussian function resembles a Gaussian/normal distribution with mean *o_s_* and standard deviation *σ*, except that the area under the curve does not sum to 1 and instead the peak value (i.e. when *x* = *o_s_*) is 1. The effect of this function is that differences between the options for adjacent steps (i.e. *o_s_* and *o_s_*_−1_ or *o_s_*_−2_) reduce received payoffs, with *σ* affecting the harshness of the penalty (Figures 1 and S4).

**Figure 2.**
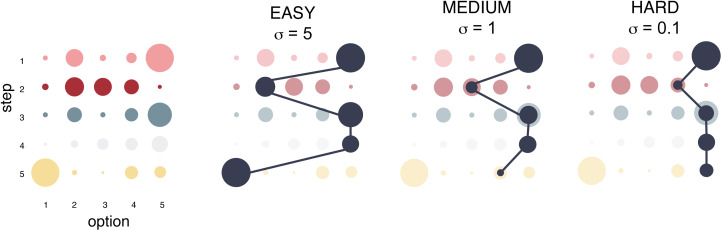
A graphical representation of how manipulating task difficulty affects the optimal solution (on a reduced version of five steps with five options, for illustration purposes). Coloured dots correspond to options, with the colour signifying the step. The size of the coloured dot corresponds to the base payoff. Chosen options are coloured black, and the size of the black dots corresponds to the received payoffs. In easy tasks steps do not restrict each other, and agents can freely explore a wide range of options. In hard tasks previous choices constrain future ones, and solutions follow much narrower paths and typically receive lower overall payoffs.

The effects of *G* and *σ* can be conceptualised as interactions between different steps: the payoff received from *o_s_* depends on *o_s_*_−1_ and *o_s_*_−2_, and *σ* is a model parameter that controls the extent of these dependencies. Our model simulates problems that are path dependent – the initial choice strongly dictate which other choices are viable later on. In practice, our implementation of difficulty translates into finding a good solution out of many, but the solution is not one choice for every step, but the entire combination of steps. A high value of *σ* translates into steps that are virtually independent, but a low value produces intense dependencies that greatly reduce payoff associated with many repertoires that deviate from the optimal path (Figures 1 and 2). Thus, *σ* modulates task difficulty. Preliminary simulations (see Figure S5) were carried out to assess how step inter-dependency (i.e. *σ*) affects difficulty, and on this basis we selected hard (*σ* = 0.1), moderate (*σ* = 1) and easy (*σ* = 5) values. Note that the dependency structure we chose implies that *o_s_* affects the payoff received from *o_s_*_+1_ and *o_s_*_+2_, but this is one-way and *o_s_*_+1_ does not affect the received payoff of *o_s_* (equations 1 and 2). As a result, payoffs from the first step are not penalised at all, and payoffs from the second step are only mildly penalised, which could mean that the first two steps can be responsible for a very large proportion of the payoffs. Allowing these steps to depend on others, however, does not change our results (Figure S6).

### 2.2. Learning algorithms

We implemented three individual/asocial learning algorithms: the ‘near-sighted learner’, the ‘mid-sighted learner’ and the ‘long-sighted learner’. These algorithms are differently able to understand how the dependency structure affects their payoffs and so can be considered to vary in ‘intelligence’. All three algorithms progress through all steps in a random order selecting the option perceived to have the greatest received payoff as they go. Variation is introduced into the population by the assumption that individuals select options for each level in a random order. Because agents always make the optimal choice given the limitations of their algorithm, assuming that learning was strictly ordered would cause all individuals within each simulation to reach the same conclusions. Relaxing this assumption, however, introduces variation contingent on the order in which levels are evaluated by each individual. While mutation (i.e. learning error) is a more common source of variation in evolutionary models, it requires assumptions about the nature, rate, scope and independence of mutations, which are sidestepped by our implementation. Nonetheless, we additionally include a form of mutation in some simulations as discussed below. Lastly, we note that the mechanics of learning are open empirical questions, thus random-ordered learning is a reasonable assumption given the lack of strong empirical justification and the importance of variation to cultural evolution.

The three learning algorithms have access to and can distinguish between payoffs (through processes like trial-and-error or mental simulation), but differ in the extent to which they take the payoff dependencies into account when estimating the effect of each option of their total payoff. The simplest algorithm, the ‘near-sighted learner’, selects the value of *o_s_* that yields the greatest received payoff. If values for *o_s_*_−1_ and/or *o_s_*_−2_ have already been selected, the ‘near-sighted learner’ takes into account how these affect the received payoff for *o_s_*. If values for *o_s_*_−1_ and *o_s_*_−2_ have not yet been selected, the near-sighted learner effectively chooses a value for *o_s_* of the basis of base payoffs alone. Note that the near-sighted learner does not consider how *o_s_* might affect the received payoffs from *o_s+_*_1_ and *o_s+_*_2_. As such it will select a value for *o_s_* that has the greatest received payoff even if it forces it to miss a high payoff value for *o_s+_*_1_, or reduces the received payoff from an already selected *o_s+_*_1_. This inability of the near-sighted learner to look ahead the dependency structure is reflected in its name.

The second algorithm is the ‘mid-sighted learner’. Like the near-sighted learner, if values for *o_s_*_−1_ and/or *o_s_*_−2_ have already been selected the mid-sighted learner considers how these affect the received payoff for *o_s_*. However, the mid-sighted learner additionally considers how *o_s_* will affect the received payoff from *o_s+_*_1_. If a value for *o_s+_*_1_ has already been selected the mid-sighted learner selects the value of *o_s_* that maximises the combined received payoff from *o_s_* and *o_s+_*_1_. Thus, it might select a low payoff value for *o_s_* in order to protect a particularly high payoff from *o_s+_*_1_. If no value for *o_s+_*_1_ has been selected the mid-sighted learner identifies the combination of values for *o_s_* and *o_s+_*_1_ that yields the greatest received payoff (taking into account any values chosen for *o_s_*_−2_ and *o_s_*_−1_) and selects the corresponding value of *o_s_*.

Finally, the ‘long-sighted learner’ is like the mid-sighted learner, except that it takes into account current or potential values of *o_s+_*_2_ as well as *o_s+_*_1_ (and *o_s_*_−1_ and *o_s_*_−2_ if they have been selected) when choosing a value for *o_s_*. Note that although the long-sighted learner is sophisticated (being able to compare the payoffs from 1000 different option combinations simultaneously), it does not necessarily make optimal decisions (see Supplementary Materials for more details).

### 2.3. Mechanisms of cultural inheritance

In addition to the three different asocial learning algorithms discussed above, we implemented three different mechanisms of cultural inheritance: ‘refinement’ ‘recombination’ and ‘combined’ (the latter being recombination followed by refinement). Refinement and recombination are both learning mechanisms that introduce innovations in the population, but the extent of these innovations differ. Following previous work we defined refinement as modifications of current solutions, while recombination was defined as combining only information that already exists in the population (Lewis & Laland, 2012; Muthukrishna & Henrich, 2016). Refinement can introduce new options in the population, while recombination cannot, and in this sense refinement is more innovative than recombination. Here we added another distinction between recombination and refinement: with refinement, agents make use of their understanding of dependencies in order to make decisions about what the best choice is, while with recombination they do not.

Inheritance is subject to error (i.e. random mutation as opposed to guided variation) such that the option chosen for a given step is randomised with probability *m*. Moreover, because inheritance is not possible in the first generation, in all cases agents in the first generation apply their asocial learning algorithm to an empty repertoire.

Agents using the refinement mechanism inherit the repertoire of a randomly selected agent from the prior generation and apply their learning algorithm (near-, mid-, or long-sighted) to it, changing any options if they identify a superior alternative. The limitations of the asocial learning algorithms still apply, so a mid-sighted agent, for example, will check if any value of *o_s_*, given the current value of *o_s+_*_1_, results in a higher total payoff.

In the recombination condition agents are randomly assigned *n_p_* agents from the previous generation to their cultural parents. For each step, the agent inherits the option from one of its parents, with the parent chosen with probability proportional to the parent's total payoff. For each step, the probability of copying that step from parent *x* is equal to the payoff of *x* divided by the sum of all payoffs of *n_p_* parents. Our choice to first select *n_p_* agents from the entire population and then copy according to their respective payoffs reflects the fact that in the real world individuals plausibly only have access to an immediate network of models and not the entire population. Nonetheless, we ran opposite ‘payoff-bias’ conditions in which agents first pick *n_p_* agents from the entire population based on their payoff, and then copy each step unbiased from this privileged set of models, but found negligible differences between the results (Figure S3). Note that, because recombination does not depend on the learning algorithm, in the recombination-only condition simulations implementing near-, mid-, and long-sighted algorithms only differ in how agents fills their empty repertoires in the first generation.

In the recombination-and-refinement case agents first recombine from *n_p_* parents, and then refine what they have inherited.

### 2.4. Parameters

Each run of the simulation involved nine parallel populations: one with every combination of learning algorithm and inheritance mechanism. The learning algorithm and the inheritance mechanism were exogenously imposed and did not change within simulations. Each run involved three separate payoff matrices, one for each inheritance mechanism. In this way, all three asocial learning algorithms were confronted with the same set of payoff matrices. We ran 500 repeat simulations for every combination of the parameter values summarised in Table 1.

**Table 1.** Parameter summary

Parameter	Values	Description
*σ*	0.1, 1, 5	Standard deviation of the penalty window (modulates task difficulty)
*n_p_*	1, 2, 5, 10	Number of randomly selected models that agents learn from
*n_a_*	10, 50, 100, 500	Number of agents in the population
*m*	0, 0.0001, 0.001, 0.01, 0.1	Random mutation rate

## 3. Results

### 3.1. General results

**Figure 3.**
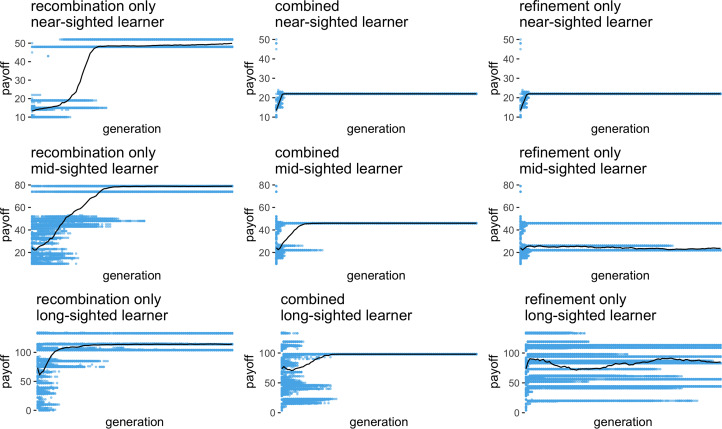
Payoffs for one run of the simulation for (a) the recombination inheritance mechanism (b) the refinement mechanism, and (c) the combined mechanism, with populations of 500 agents each, *n*_p_ = 2, *σ* = 0.1, over 100 generations, and the same payoff matrix. Every point indicates the payoff for one individual, and the black line follows the population mean. In this example, near-sighted learners converge quickly on a suboptimal solution when using the refinement and combined mechanism, but increase in performance over time and converge on a high payoff solution with recombination. Mid-sighted learners behave similarly. Long-sighted learners converge on a good solution with recombination only, but converge on a suboptimal solution when using the combined mechanism, and never reach convergence with the refinement only.

Across all parameter values, inheritance mechanisms and learning algorithms, payoffs typically increased over time as agents improved upon their inherited repertoires (Figure 3). However, there was considerable variation between simulation repeats, both in terms of the payoff distribution and in terms of the pattern of performance over time (Figure S7). As mentioned above, every new run used a new reward matrix, and given the nature of exponential distributions that base payoffs are drawn from, reward matrices are highly variable, both in terms of how easy successful repertoires are to find and in the range of payoffs. To account for this, hereafter we present normalised payoffs, whereby the payoff of each agent is scored relative to the average payoff of 10,000 long-sighted learners without cultural inheritance. Ideally, we would be able to measure the performance of a ‘perfect’ learner on every payoff matrix and normalise the payoffs relative to this maximum. However, this would mean iterating through every possible option for every step – for a 10 by 10 matrix this translates to 10^10^ options, which renders this task computationally unfeasible. Such calculations are easily computable for matrices of 5 steps × 5 options, and we ran additional simulations using such smaller matrices with payoffs normalised according to the optimal payoff and found very similar results to those presented here (Figure S8).

### 3.2. Asocial learning and cultural inheritance

**Figure 4.**
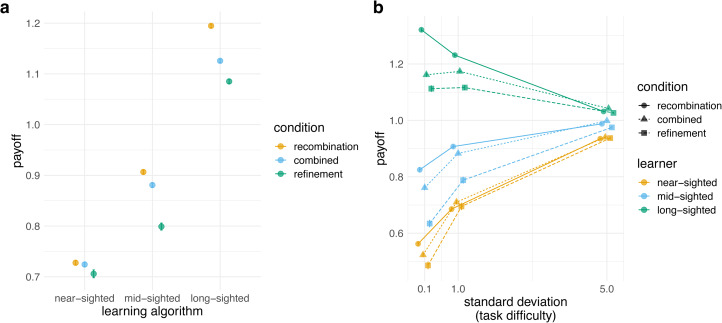
(a) Average normalised payoff for each learning algorithm relative to the average 10,000 individual long-sighted learners, where the colour indicates the mechanism. (b) The effect of task difficulty: normalised payoff for each mechanism relative to average payoff of 10,000 individual long-sighted learners, where the shape indicates the condition and the colour indicates the learning algorithm foresight, across three standard deviation values (0.1 for difficult tasks, 1 for medium, and 5 for easy). The points plot the values in the final generation (after 60 generations), averaged over 500 repeated simulations, averaged over all other parameter combinations with bars indicating two standard errors.

Across all three inheritance mechanism conditions, long-sighted learners achieved higher performance than mid-sighted learners, who did better than near-sighted learners (Figure 4a). Unexpectedly, the best performance was achieved by long-sighted learners using recombination alone, which out-performed the same learners with refinement alone or recombination and refinement combined. This was not the case with the other learning algorithms: for mid-sighted learners, recombination was comparable with the combined mechanism, while refinement alone was worse. For near-sighted learners, performance was similar across all three conditions.

There was a clear interaction between the value of the standard deviation and the foresight ability of the learning algorithm (Figure 4b). When the task was easy, all learners achieved comparable payoffs across all learning mechanisms. When the task was difficult, however, long-sighted learners achieved much higher payoffs. For example, when the task was easy, long-sighted agents using the combined mechanisms did 10% better than near-sighted agents using the same inheritance mechanism, but when the task was hard, long-sighted agents did 55% better.

When the task was difficult, long-sighted agents achieved higher payoffs, yet particularly so when learning through recombination (this difference was robust to mutation, see Figure S10). Long-sighted agents learning using recombination alone performed much better that the long-sighted agents learning through the combined mechanism. When the task was easy, long-sighted agents using the combined mechanism performed 2% better than the same agents using recombination alone, but when the task was difficult recombination resulted in 12% better performance than recombination and refinement combined.

Compared with the effects of learning algorithm, the influence of the demographic parameters (number of cultural parents and total number of agents in the population) was small, although demography affects recombination more so than the other conditions (Figure S9). Mutation (i.e. imperfect learning, erroneously replacing a copied step with a different one) only considerably affected the results when very high (Figure S10): we saw a considerable dip in payoffs for *m* = 0.1, which for a repertoire of 10 steps translates into an average of one mutated step every generation for every agent. Performance under recombination-only suffered much more because of mutation than performance in the other inheritance mechanisms, suggesting that refinement provides a mechanism that mitigates copying errors.

### 3.3. Cumulative cultural evolution

To test for cumulative cultural evolution (the increase in payoffs over generations owing to cultural inheritance), we normalised the payoffs relative to the *average* individual learner in the first generation within each learning algorithm (i.e. near-sighted payoffs relative to the average individual near-sighted learners, mid-sighted payoffs relative to the average individuals mid-sighted learners, etc.). This allowed us to investigate whether population performance increased over multiple generations.

**Figure 5.**
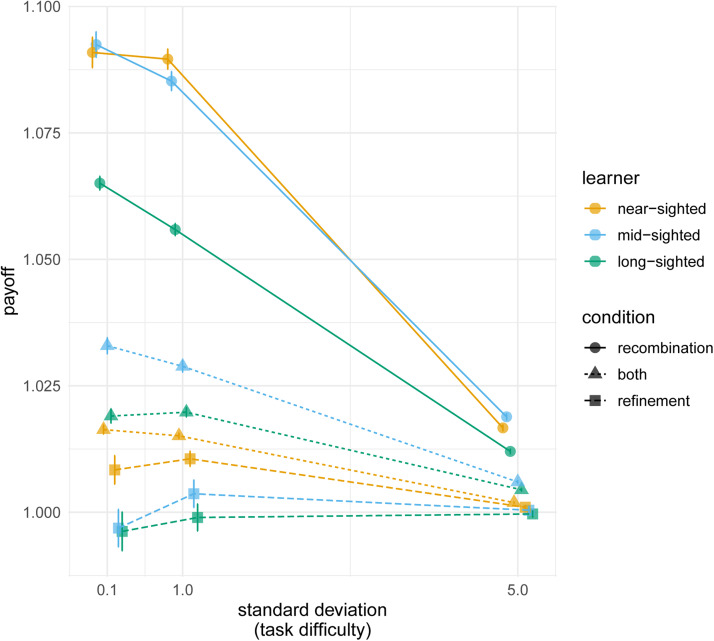
Payoffs across task difficulty relative to performance of average individual learners in the first generation within each learning algorithm. The shape indicates the mechanism and the colour indicates the learning algorithm foresight, across three standard deviation values. The points plot the values in the final generation (after 60 generations), averaged over 500 repeated simulations, averaged over all other parameter combinations, with bars indicating two standard errors.

Results show that indeed cumulative cultural evolution is taking place: by the final generation of the simulations, all average payoffs were higher than 1, above what a typical agent can learn on its own (Figure 5). Cumulative cultural evolution was most pronounced when agents used recombination alone, and this was particularly true for individuals with less foresight. There was more improvement when the task was difficult. These results indicate that when the task is easy and the agents smart enough, a single agent learning alone achieves near-ceiling performance, but for harder tasks (or when agents have less foresight) individual performance suffers and the iterative progress of intergenerational learning has more pronounced benefits.

These results might seem to suggest that near-sighted learners, who achieved the most accumulation, performed best. However, recall from the above section that their overall performance was considerably worse than the mid- and long-sighted learners. Thus, while near-sighted learners are most improved by cumulative cultural evolution, long-sighted learners show the greatest payoffs both in the first generation and after subsequent cumulative cultural change.

### 3.4. Diversity

**Figure 6.**
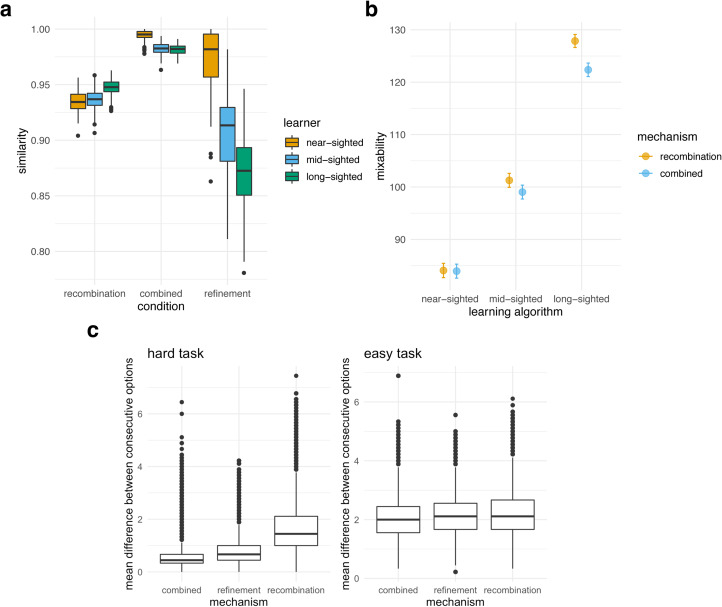
(a) Distribution of repertoire similarity at the end of 60 generations, averaged over all agents in each of 200 simulation repeats, and averaged over all parameter combinations for near-, mid- and long-sighted learners, for the three mechanisms. (b) Mixability at the end of 60 generations relative to the random baseline for near-, mid- and long-sighted learners averaged over 200 runs and over all other parameter values (*σ*, *n*_a_ and *n*_p_), for the recombination and combined mechanisms. (c) Distribution of repertoire uniformity (measured as the mean difference between consecutive options) for simulations in the three mechanisms, for hard and easy tasks, after 60 generation and 200 runs. A high difference translates into lower uniformity – recombination maintains more varied repertoires, and more so when the task is hard.

These results hint at one apparent contradiction: asocial intelligence is both good (long-sighted learners outperform near-sighted learners) and bad (recombination performs best). Asocial intelligence, even when used concurrently with recombination in the combined condition, results in suboptimal performance when compared with recombination only. We set out to understand how recombination can produce the best performance despite making minimal use of asocial intelligence. We hypothesised that recombination is useful under this variety of conditions because recombination maintains enough variation to allow the population to jump between local peaks, while refinement drives population to converge too quickly on similar repertoires and lose any variation that would push the population out of suboptimal peaks. To measure diversity we developed a metric of cultural similarity. The similarity of two repertoires is the proportion of steps for which they have identical options, and population similarity is the average similarity between all pairs of agents. Final generation similarity differed dramatically across inheritance mechanisms: populations using the combined mechanism converged on highly similar repertoires, regardless of the learning algorithm used (Figure 6a). Populations using refinement only showed large variation in repertoires, particularly with the more complex learning algorithms, probably caused by the fact that individual refinement results in separate lineages within one population. Recombination produced an intermediate level of diversity. This pattern was consistent across a variety of measures of cultural diversity (Figure S11).

We also measured diversity *within* repertoires as ‘repertoire uniformity’: the average numerical difference between consecutive steps across all agents in the population. Uniform repertoires are those in which consecutive steps have similar option values (e.g. [3, 3, 4, 4 …]) while diverse repertoires are those in which consecutive steps have highly different options (e.g. [3, 8, 1, 10 …]). When the task is hard the dependency structure incentivises relatively uniform repertoires (i.e. similar values for each element in the repertoire), because large differences between consecutive steps are penalised (Equation 1). We found a difference in repertoire uniformity between mechanisms and, importantly, this difference was modulated by task difficulty (Figure 6c). Generally, repertoires under the recombination mechanism were less uniform than repertoires under the other two mechanisms – populations under the combined mechanism often converged on a single repertoire consisting of identical options, even when this was suboptimal (Figure S12), and the difference between inheritance mechanisms was much higher in difficult tasks.

Taken together, these results suggest that through refinement the population converges on different lineages consisting of highly uniform repertoires, yet through recombination populations maintain more variation, both at the population and at the repertoire levels. In the combined mechanism, the recombination step disrupts the lineage formation that takes place with refinement only, but the refinement step still reduces diversity, producing a single, low-diversity lineage.

### 3.5. Mixability

A final outstanding question concerns how recombination only can achieve the best performance despite the fact that it maintains diversity, when hard tasks require very precise combinations of options for success. We developed a measure of the utility of the variation in the population, which we termed ‘mixability’. We hypothesise that via the repertoire-level selection inherent to copying from parents proportional to their payoff, recombination filters out options that do not mix well with other options prevalent in the population, maintaining only options that combine well with each other and result in high payoffs. Mixability is thus a population-level measure that quantifies how well a collection of repertoires mixes and is suitable for recombination. Increased mixability is beneficial for recombination because it means that any combination of options will probably achieve a reasonable payoff, while a population low in mixability could, for instance, consist of multiple high-payoff repertoires, but nonetheless be unlikely to result in any high-payoff repertoires in the next generation.

We calculated mixability at the end of the final generation of each simulation, after 60 generations. In order to calculate mixability, for each step we used only the options still present in the population after the 60th generation. We generated 1000 repertoires by sampling randomly, for each step, from the options still available in the population for that step. This sampling was done regardless of the frequency of the options in the population. For example, if the only two repertoires available in the population at the end of a simulation were [1, 2, 3, 4, 5] and [6, 7, 8, 9, 10], one random reshuffling would consist of either option 1 or option 6 with equal probability for the first step, 2 or 7 for the second step, etc. Mixability is calculated as the average payoff of the 1000 repertoires generated this way. If our hypothesis is correct, then shuffling options in this manner will result in more effective repertoires in the recombination condition.

We ran 200 additional simulation repeats for the recombination-only and combined mechanisms and measured mixability at the end of the simulations. Over time, populations increased in mixability (Figure 6b), and mixability was higher for recombination than for the combined mechanism, particularly so for long-sighted learners. This confirms that recombination filters out repertoires less effective at mixing with other repertoires in the population. Note that recombination achieves this despite having more population-level and repertoire-level diversity than the combined mechanism.

## 4. Discussion

We found that, in the context of this model of a complex cultural system, the best payoffs are achieved through a fine balance between individual intelligence, cultural inheritance mechanism, task difficulty and demography. When the task was easy, performance was comparable across inheritance mechanisms and learning algorithms because even individual agents with little understanding of the task structure could find a reasonable solution. When the task was difficult, however, the foresight of the learning algorithm became crucial to finding high-payoff solutions.

The best performance was achieved by populations of intelligent individuals who made minimal use of their intelligence and relied on recombination only, and the superiority of recombination was particularly apparent on difficult tasks. This result was due to the different types of selection that recombination and refinement rely on. Recombination uses a ‘repertoire-level selection’, where steps are learned according to the overall payoff of the parents’ repertoires. Note that although recombination necessarily takes account of the effects of interactions, it does not attempt to understand the interactions themselves. Refinement, however, uses a ‘step-level selection’ or guided variation (Boyd & Richerson, 1985), where individuals rely on their understanding of the task to update steps if alternative options are perceived as better. This approach takes account of between-step interactions, but only as far as the learner can understand them. When the task is easy, local knowledge is enough for individuals to find successful solutions and (marginally) outperform agents using only recombination, but as the task becomes more difficult, refinement's imperfect grasp on step interactions struggles to identify successful combinations. In this case, recombination, relying on repertoire-level selection, provides a more effective way of pruning out ineffective step combinations.

We note the somewhat counterintuitive result that, although the complexity of the learning algorithm is a major determinant of success, on all but very easy tasks the best performance is achieved when agents make minimal use of their intelligence and rely instead on recombining the output of an initial generation of innovators. This highlights that, although high-quality information produced by intelligent agents is valuable, so is the strategic trade-off between intelligence and reliance on the population-level process of payoff-biased recombination.

As a caveat, we note that even the least intelligent agents we considered are sophisticated in the sense that they have access to and can accurately distinguish between payoffs through mechanisms like mental simulation and trial-and-error learning. The key factor that differentiated the learning algorithms was their understanding of dependencies, which in effect allowed the intelligent learners to compare many more options (up to 1000 at any one time, vs. 10 for the least intelligent agents). Thus, our implementation of intelligence is more closely associated with the ‘scale’ or ‘scope’ of an agent's understanding of the task, as opposed to their accuracy in perceiving payoffs. Such an implementation is useful given our research goals and the type of modular task investigated here.

We considered two cases: first-generation innovation followed by recombination only, or recombination and refinement by every individual. However, there is evidence that optimal results are often achieved through a diversity of learning strategies rather than homogenous top-performers – progress is often achieved by a mix of risky innovators and refiners (Hong & Page, 2004; Miu, Gulley, Laland, & Rendell, 2020; Thoma, 2015), and in partially connected populations that prevent a single idea from becoming ubiquitous and allow for diversity of solutions (Derex & Boyd, 2015; Derex et al., 2018). What is more, different population structures prove beneficial for problems of different difficulties (Lazer & Friedman, 2007). The current work set out to compare inheritance mechanisms, but we expect that if we considered intermediate cases where the population is a mix of recombiners and refiners, the best performance would be achieved through a combination of the two. Similarly, we were interested in what strategies prove useful for difficult problems. Further work should determine what be optimal combination is, and how that combination varies across types of problems.

How is recombination, a process entirely ignorant of option-level payoffs, able to achieve such high performance? Our analyses of diversity, uniformity and mixability suggest that the answer lies in the kind of variation the different inheritance mechanisms produce. Populations learning through recombination maintain a wider variety of less uniform repertoires, yet this variation is not random. Recombination converges on repertoires that can be recombined with others in the population without suffering severe payoff decline (‘good mixers’). Mixability is not something that refinement considers and, when interactions are harsh, refinement improves payoffs in the short term, but introduces poorly mixing repertoires that, in the combined condition, decrease payoffs in the next generation. This result is related to what Livnat et al. (2008) suggest as one consequence of sexual reproduction with genetic recombination. The authors did not focus on selection for fitness, but instead investigated how selection for mixability of genes is affected by sex. They found that genetic recombination favours alleles that do well across a variety of genetic contexts, at the expense of alleles that rely on specific conditions, even when their potential payoff is superior to generalist alleles. However, in Livnat et al.'s model alleles vary in how much their fitness depends on context, and thus some alleles are objectively ‘good mixers’; in our model good mixers are only defined relative to the composition of the population, and mixability is achieved by the population converging on a set of similar options.

This result – that the best payoffs are produced by intelligent agents who rely on payoff biased recombination instead of their own intelligence – may help resolve an ongoing debate over the nature of cultural adaptation. Many theories have suggested that it is human individual intelligence that is responsible for our ecological success, proposing that humans possess an ‘improvisational’ (Pinker, 2010) or ‘technical’ (Osiurak et al., 2016) intelligence that allows us to engineer solutions to ecological problems, then shared by cultural inheritance. However, such theories are countered by work showing that humans rarely understand the details of cultural adaptations (Boyd, 2017; Boyd, Richerson, & Henrich, 2011; Henrich & Henrich, 2010) and that it is properties of human social networks, rather than individual brains, that have driven cultural adaptation (Muthukrishna & Henrich, 2016). From this perspective our success stems from the population level process of cultural adaptation, which can seem remarkably detached from our individual intelligence (Boyd, 2017; Henrich, 2016). Our results offer a middle ground by which both accounts contribute: cultural adaptation does rely on sophisticated individual intelligence, but the greatest success is achieved when the bulk of the evolutionary process is entrusted to payoff-biased transmission. Thus, effective cultural adaptation involves both complex individual cognition and ‘blind’ cultural transmission.

We found a small effect of demography – agents using recombination achieved higher performance when learning from more models, and in larger populations, while the combined mechanism was unaffected. This is in line with previous work identifying population size as a moderator of cumulative cultural evolution (Henrich, 2004), and the mechanism suggested by Henrich could very plausibly be underlying the effect witnessed here – as the number of models and populations increase, so does the probability of effective solutions in the population that individuals would copy. In Henrich's model agents copy the best solution, but we expect that the mechanism of pruning ineffective step combinations discussed above, in practice, achieves a similar result, leading the population to converge on the best solutions. The effect of demography in our data is modest compared with the effect of learning algorithm and inheritance mechanism, suggesting that demography might not be as critical as previously thought. Rather, our data suggest that the success of CCE largely relies on the learning processes (e.g. innovation, recombination). Moreover, our findings suggest that such mechanisms are differentially sensitive to demographic factors. For instance, population size could strongly affect an unguided population size process like recombination, while having little effect on processes relying more heavily on individual cognition, like refinement.

We found evidence of cumulative cultural evolution in all conditions: the populations increased in performance over time, ending well above the expected performance of an individual learner, and this effect was larger for harder tasks. The minimal scope of CCE on easy tasks reflects that the populations start close to ceiling performance, so there is little progress for cultural evolution to achieve. This in part reflects our task design, where nothing is stopping an innovator in the first generation from immediately adopting the optimal repertoire. In other existing work this is not possible, as innovation is such that the optimal trait can only be achieved over multiple generations (Derex et al., 2018; Mesoudi, 2011).

Nonetheless, the CCE observed in our simulations was modest in the sense that populations only exceeded the performance of typical intelligent individual learners by a small amount (typically 10% over the baseline). Real-world human culture evolves in an open-ended fashion: new solutions are invented for old problems, and new niches open up, creating new problems. Our task, however, was designed to include a finite number of steps and options, characterised by an optimal score that could not be surpassed. We were interested in designing a task of high difficulty, as problems solved by human culture often are, and did not constrain our learners’ initial abilities, as cultural evolution models often do. Long-sighted learners in this context had considerable knowledge about the dependency structure, thus they could achieve high payoffs immediately, leaving relatively little for cultural transmission to contribute. Additionally, in our model dependencies impose penalties on the total payoff but cumulative cultural evolution often results in more efficient or more complex traits. Our task accurately captures the dynamics we were interested in, allowing us to establish the relative benefits of different cultural inheritance mechanisms, but if we were specifically interested in modelling particular dynamics of cumulative improvement, an open-ended task that would allow for infinite improvement would perhaps be more suitable. Here, by imposing substantial structure to our task we attempted an explicitly mechanistic, partly cognitively realistic implementation of learning that has been much called for (Claidière, Scott-Phillips, & Sperber, 2014; Heyes, 2017), but naturally this first attempt will have to be followed up by considerably more work in order to establish its generality.

In addition, we should recognise that cultural adaptation here does not necessarily attempt to produce what the best individual performance could be. With recombination, selection must strike a balance between payoff and mixability: if the highest payoff repertoire is a poor mixer, it will not be favoured by selection. Therefore, the top scoring repertoire in the initial generation may be reshuffled into a low-paying repertoire in future generations through recombination. That recombination can introduce variation harmful to performance might suggest that refinement should produce the best outcomes, but our data clearly show that its hill-climbing approach is only effective when the task is very easy.

Our results together identify a package of key, inter-dependent ingredients, that must all be present for pronounced cumulative cultural evolution to occur. This view is in line with current large-scale theories that explain human ecological success not as the result of any one decisive factor, but of complex co-evolution of genes and culture involving numerous components, each necessary (Henrich, 2016; Laland, 2017). First note that, here, cumulative cultural evolution is minimised when populations are faced with easy tasks, because simpler problems can be quickly solved by individual intelligence alone. Second, on hard tasks, the greatest performance relies on a counterintuitive combination of asocial and social learning: agents must be asocially highly intelligent but rarely use this intelligence. Individual intelligence, as we implemented it, achieved poorer performance than recombination alone. Naturally, there could be other forms of complex interactions between recombination and individual intelligence that we have not considered (e.g. considering all permutations of options among all demonstrators), but we cannot comment on those. Third, social learning must be highly faithful, as CCE suffers once cultural mutations are introduced (Figure S10). Finally, individuals must be embedded in a population that is sufficiently large/connected.

Collectively these results present a chicken-and-egg problem: pronounced CCE depends on a conjunction of many traits, yet their payoffs may be low until all traits are present. Thus, the conjunction of all these factors is expected to be rare, which may help explain why human-like CCE is unique to our species. Many of these criteria can be seen in the differences between humans and other great apes. For instance, chimpanzee cultures have been argued to be limited to behaviours that are sufficiently simple to be readily reinvented by individuals (Tennie, Call, & Tomasello, 2009, 2012). Experimental studies of human social learning typically document that most individuals rely extensively on social learning while only a minority tend to rely on their own decisions (Efferson, Lalive, Richerson, McElreath, & Lubell, 2008; Molleman, van den Berg, & Weissing, 2014; Rendell et al., 2011). Finally, there is a rich literature documenting our species’ remarkable capacity for high-fidelity social learning when compared with other primates (Dean et al., 2012; Hopper, Lambeth, Schapiro, & Whiten, 2008; Tomasello, 1999; Whiten, McGuigan, Marshall-Pescini, & Hopper, 2009), while the scale and inter-connectedness of human populations greatly exceed those of almost all other species (Richerson & Boyd, 1998). Precisely how our species acquired this package of traits while no other species has remains unclear.

Finally, if different learning processes like recombination and refinement are involved in CCE, then we should acknowledge that different processes may be more relevant in different domains, and that these processes would interact with moderating factors differently. For example, in a very different study involving creating advertisements, Ren, Nickerson, Mason, Sakamoto, and Graber (2014) found that refinement results in more creative and more effective ads than recombination, suggesting domain is important. Indeed, in an unrelated study investigating the relationship between population size and the complexity in folktales, the authors found mixed results (Acerbi, Kendal, & Tehrani, 2017) – a positive relationship between population size and complexity measured as the overall number of folktale types, but a negative relationship with complexity measured as the number of tale motifs. The authors suggest that this relationship may be domain dependent. For a functional domain like technology, where faithful replication is necessary for tool functionality, we would expect a clear relationship with population size, as the more individuals there are in the population to copy faithfully, the higher the chance is that the population will successfully transmit and improve the information. Yet in a domain like folktales, where cultural traits are free to change, more heavily influenced by individual cognitive biases, we should see a weaker relationship with population size, as complex traditions should be easier to maintain in small and large populations as long as they are guided by cognitive biases. Here we go a step beyond and propose that, for instance, a functional domain like tool making, in which the relationship between payoff and traits is not obvious and in which replication is necessary to maintain functionality, should rely more heavily on an unguided process like recombination here, while a domain like folktales would rely less on recombination and more on a process that involves individual cognition. Therefore, more detailed mechanistic understanding of the processes involved in cumulative cultural evolution would not only lead to a thorough understanding of this phenomenon, but could also explain some of the disagreements in the literature and, ultimately, contribute to a comprehensive understanding of human culture.

## Supplementary material

For supplementary material accompanying this paper visit http://dx.doi.org/10.1017/ehs.2020.42. click here to view supplementary material

## Data and code availability

The data and the code used for analyses are available at https://osf.io/h79z2/?view_only=14a42c808d5b4e89a267bf294ece9dd1
