# Supplementary material for: Cultural adaptation is maximised when intelligent individuals rarely think for themselves
Source: Evol Hum Sci. 2020 Aug 10;2:e43. doi: 10.1017/ehs.2020.42 (PMC10427482; doi:10.1017/ehs.2020.42)
Supplement: Supplementary file 1 [file S2513843X20000420sup001.pdf]

SUPPLEMENTARY INFORMATION

Cultural adaptation is maximized when intelligent individuals rarely think for themselves

Elena Miu, Thomas J.H. Morgan

Contents:

Supplementary Figures 1-12

Supplementary material

1. Number of generations

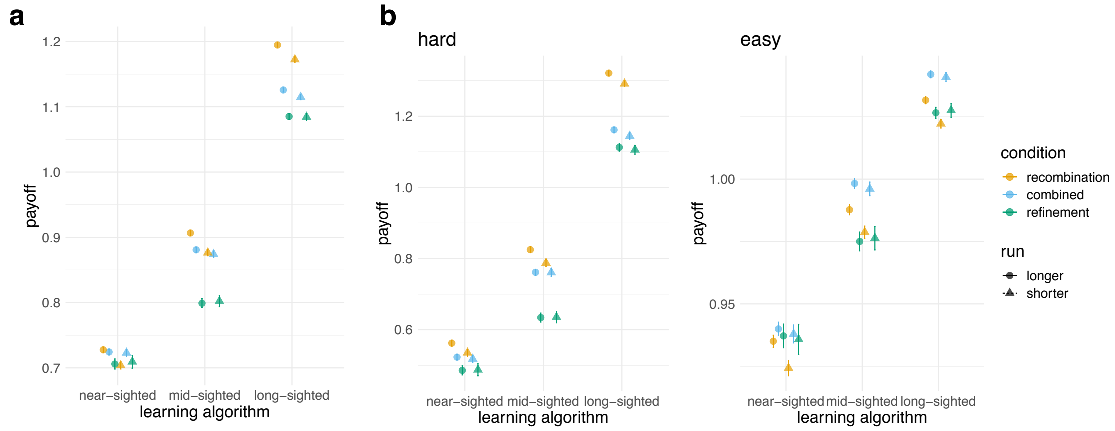

Figure S1 – comparison between shorter runs (30 generations) and longer runs (60 generations). (a) Average normalised payoff for each learning algorithm relative to the average 10,000 individual long-sighted learners, where the colour indicates the mechanism, and the shape the length of the run. (b) The effect of task difficulty: normalised payoff for each mechanism relative to average payoff of 10,000 individual long-sighted learners, where the colour indicates the learning algorithm, and the shape the length of the run, for easy tasks (σ=5) and hard tasks (σ=0.1). The points plot the values in the final generation, averaged over 500 repeated simulations, averaged over all other parameter combinations with bars indicating two standard errors.

2. Payoff matrix distribution

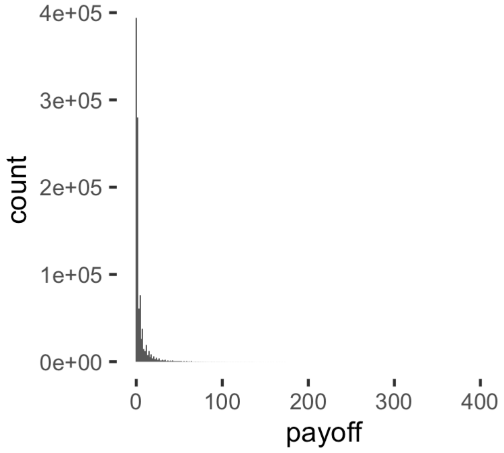

Figure S2 – 1,000,000 samples drawn from a typical payoff distribution. The options for each step would be associated with payoff distributions like these.

3. Learning algorithms do not always find the optimal solution

Note that although (*i*) o_s only affects the received payoffs from o_{s+1} and o_{s+2} and (*ii*) the long-sighted learner can take this into account, this does not mean the long-sighted learner makes optimal decisions. This is for two reasons. First, like all learning algorithms, the long-sighted learner progresses through the steps in a random order and this may prevent it from fully utilising its ability to recognize interactions. For instance, if it were to progress "backwards" through the steps (i.e. from step n_s to 1) it could not use its ability to plan ahead by choosing the value for o_s on the basis of the perceived best combination of o_s, o_{s+1} and o_{s+2}. Second, even if it progresses through steps in order (i.e. from step 1 to n_s) it can make suboptimal decisions because although it can choose a value for o_s on the basis of what appears to be the best possible combination of o_s, o_{s+1} and o_{s+2}, it cannot be sure this is optimal without knowing how the values for o_{s+1} and o_{s+2} go on to affect o_{s+3} and o_{s+4}, and so on.

Long-sighted learners are not guaranteed to make optimal decisions although they can choose values for the current step o_s based on the best combination of o_{s+1} and o_{s+2}, because what appears to be the best combination may not be so, depending on the structure of the payoff matrix.

For example, depending on how much foresight an agent has and depending on how constrained the steps are, an agent would underperform on this matrix:

[1, 1, 1, 1]

[3, 1, 1, 1]

[10, 0, 0, 0]

[0, 0, 0, 50]

A long-sighted learner will, first, check all the combinations of options for steps 1, 2, and 3, and pick, for the first step, the option that, out of all the combinations, achieves the highest combined payoff. In this case, in a medium difficulty task (σ=1), the best combination of the first three options is o_1=1, o_2=1, o_3=1, resulting in a collective payoff of 14.

[1, 1, 1, 1]

[3, 1, 1, 1]

[10,   0,   0,   0]

[0,   0,   0, 50]

Next, it will assess every combination of options for steps 2, 3, and 4, and choose the best option, given that it has already picked option 1 for step 1. In this case, the best combination of steps is o_2= 1, o_3=1, o_4=4, with a collective payoff of 13.00617. Note that because the agent's first choice was option 1, this highly penalised the payoff of option 4 for the last step. This agent will go on to choose option 1 for step 3, and option 4 for step 4, resulting with the final repertoire [1,1,1,4] with a total payoff of 14.00617.

The following show the final received payoffs, with the choice in red:

[1,   1,   1,   1]

[3,   0.6065, 0.1353, 0.0111]

[10, 0,   0,   0]

[0,   0,   0,   0.00617]

The best repertoire, in this case, is [4,4,4,4], with a total payoff of 52. Because the long-sighted learner makes its first choice just based on the first three steps, it takes a sub-optional path that it cannot recover from.

4. Payoff biased recombination

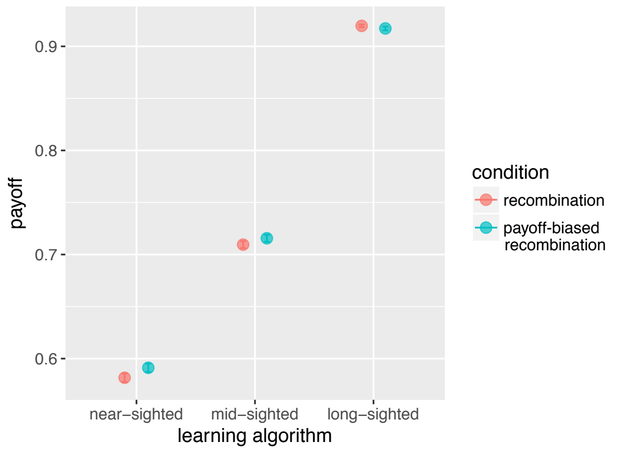

Figure S3 – comparison between the recombination learning mechanism implemented (agents pick *x* random models, then copy step by step proportional to the models' payoff) and "payoff biased" recombination, wherein agents pick *x* models based on their payoff, then copy step by step at random. Normalised by best individual in the first generation. Average payoff for the two mechanisms over each of the three learning algorithms, averaged over 300 repeat simulations, and all other simulation parameters. We found practically no difference between the two conditions.

5. Modulating task difficulty

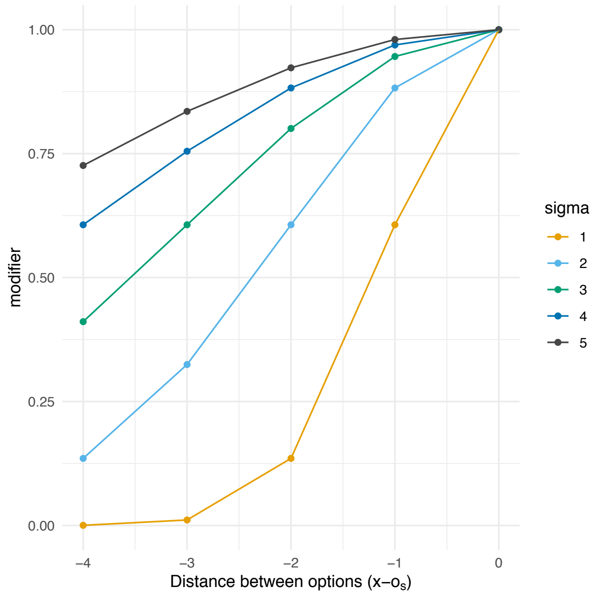

Figure S4 - the effect of sigma on payoffs – penalties as a function of the difference between subsequence options. The higher the difference between current and previous option, the higher the penalty to payoff.

We explored a series of values for the standard deviation in order to understand how step dependency affects difficulty (Fig. S5). We used the payoffs from each of the learning algorithms to verify that our way of modulating difficulty is effective – if the task is easy, then all three learning algorithms should achieve similarly high payoffs, while if the task is difficult we expect long-sighted learners to perform much better than near-sighted learners. For this explorative analysis we assumed no intergenerational learning. Instead, each population consisted of one generation of individual learners building a repertoire, and we compared payoff distributions across near-, mid-, and long-sighted learners. As expected, when the standard deviation was high we saw little improvement between near-, mid-, and long-sighted learners, indicating that the task was easy and did not require advanced knowledge of the dependency structure. When the standard deviation was low, however, there was a high increase in performance between each learning algorithm, suggesting that learners with limited information about the dependency structure struggled to keep up with long-sighted learners who had full knowledge, which we would expect to be the case for a difficult task.

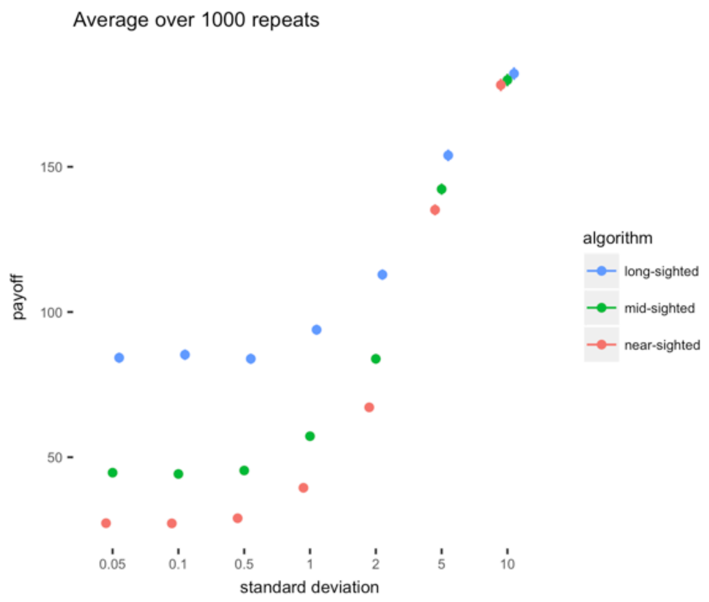

Figure S5 – average payoffs and standard errors for 1000 repeats of agents who learn individually using each of the three learning algorithms, for varying values of the standard deviation. For each of the 1000 repeats a new payoff matrix is generated, and for every payoff matrix a near-, mid-, and long-sighted learner learns a repertoire for each of the 7 values of the standard deviation.

6. “Donut” dependencies

In the dependency structure we present in the main paper, payoffs from the first step are not penalized, and payoffs from the second step are penalized less, which can skew the payoff distribution. This is partly intentional – we were interested in problems characterized by path dependency, where initial choices affect the possible choices made later on – and partly for convenience. In order to check that this choice does not skew the results in any meaningful way, we have run additional simulations using a set-up in which step dependencies are “donut” shaped, i.e. the last two steps affects the payoffs from the first and second step. We ran a subset of the simulations under these conditions and have found very similar results to the results presented in the main paper, and no qualitative differences

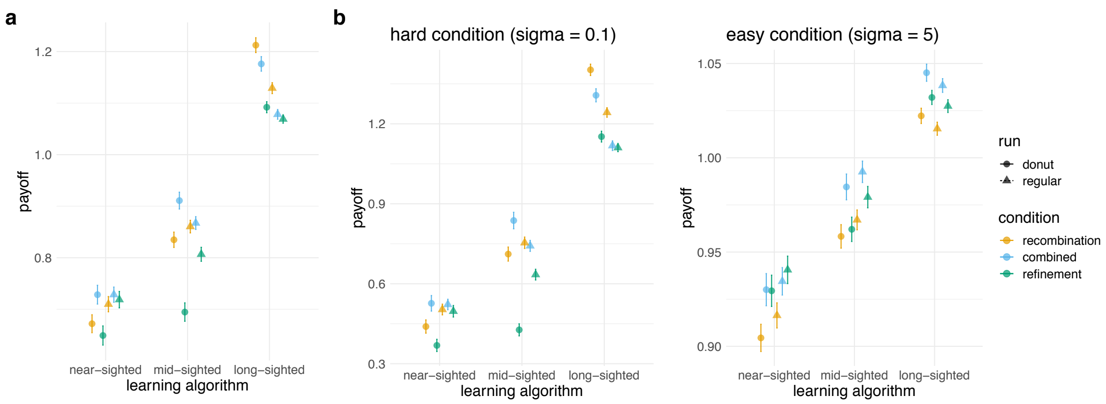

Figure S6 – comparison between regular simulations presented in the main paper and “donut” dependencies, where the last two steps affect the first two. (a) Average normalised payoff for each learning algorithm relative to the average 10,000 individual long-sighted learners, where the colour indicates the mechanism, and the shape the dependency structure. (b) The effect of task difficulty: normalised payoff for each mechanism relative to average payoff of 10,000 individual long-sighted learners, where the colour indicates the learning algorithm, and the shape the dependency structure, for easy tasks (σ=5) and hard tasks (σ=0.1). Results from a subset of parameters (n_m = 2). The points plot the values in the final generation (after 30 runs), averaged over 200 repeated simulations, averaged over all other parameter combinations with bars indicating two standard errors. We found no qualitative differences between the two conditions.

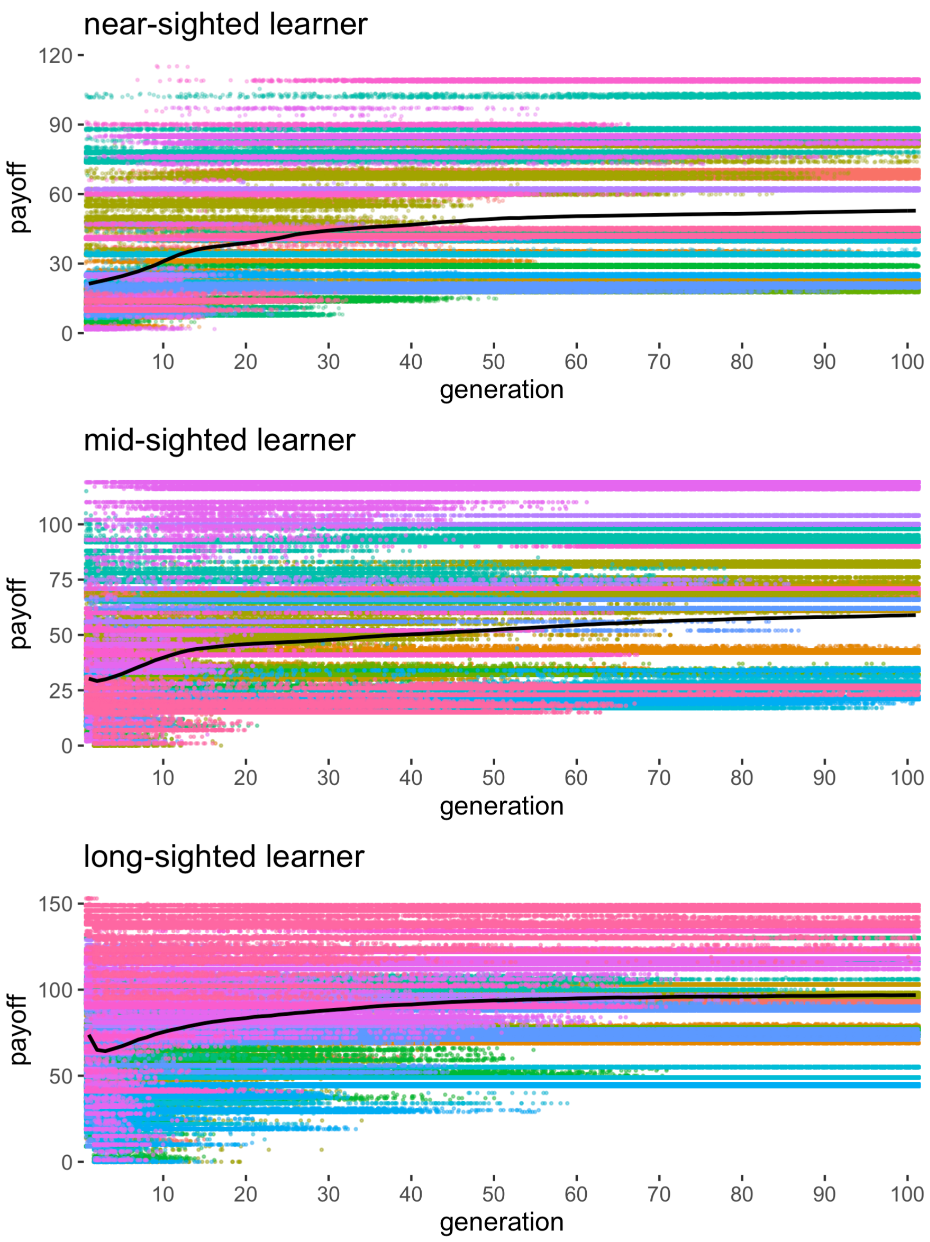

Figure S7 – between-run variability: payoffs for 15 runs of the simulation for the recombination only mechanism, with populations of 500 agents each, $n_p = 2$, $\sigma = 0.1$, over 100 generations. Every point indicates the payoff for one individual, with different colours for different runs of the simulation, and the black line follows the mean payoff for all runs.

7. Perfect learners

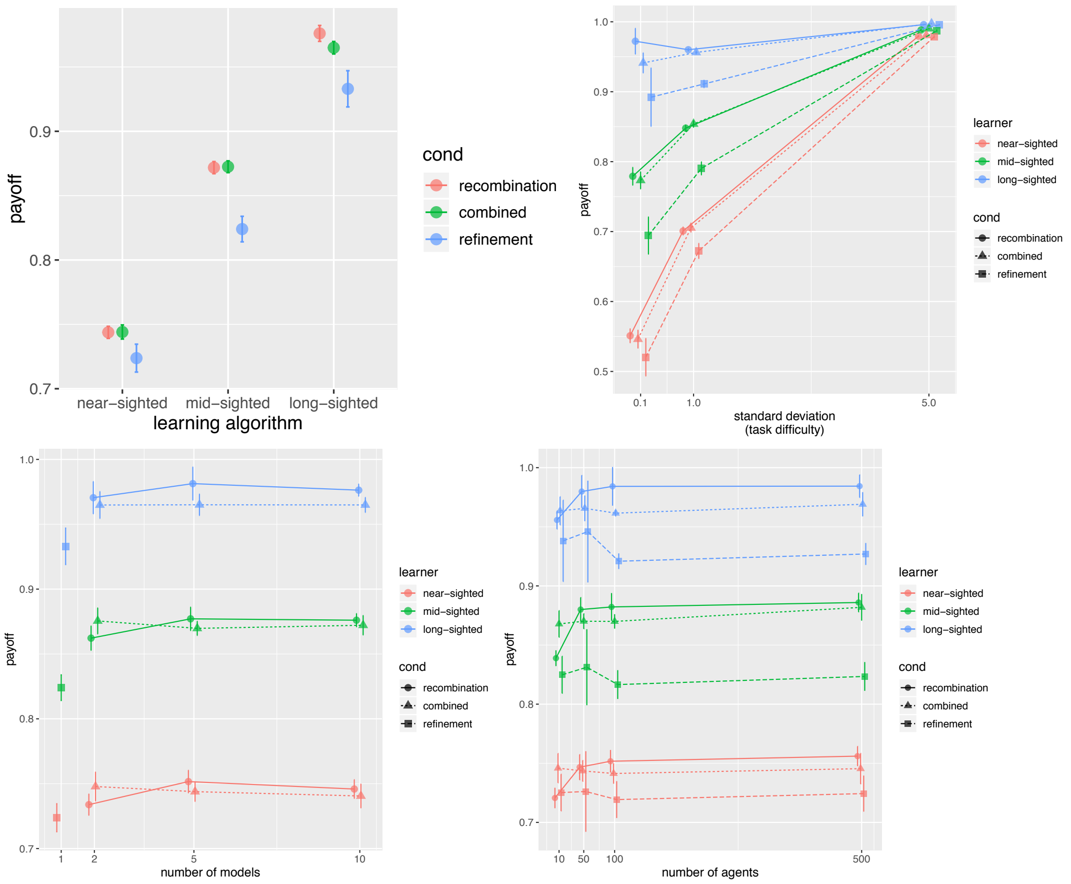

Figure S8 – (top left) overall results, (top right) the effect of task difficulty, (bottom left) the number of models and (bottom right) the number of agents on a grid of 5 steps, each with 5 options: normalised payoff for each mechanism relative to the *optimal solution*, with the shape indicating the mechanism and the colour indicating the learning algorithm. The points plot the values in the final round (after 30 generations), averaged over 500 repeated simulations, averaged over all other parameter combinations, with bars indicating two standard errors.

8. Demographics

The number of models had a very minor effect on payoff for the combined mechanism for any of the three learning algorithms (Fig. S8). All learners using refinement alone performed worse than the combined learners, therefore recombining information from more sources and then refining is a more effective strategy than simply refining. When recombining alone, however, the number of models matters more and performance increased somewhat with the number of agents recombined from, although this effect diminished as the number of models increased beyond 5. There was an interaction between inheritance mechanism, learning algorithm, and the number of models on performance: Long-sighted learners consistently (i.e. regardless of the number of models) performed better in the recombination only mechanism than in the combined mechanism, but with mid-sighted learners the recombination-only mechanism was superior to the combined mechanism only when the number of models was 5 or more, and for lower values the combined mechanism produced the best performance. With near-sighted learners the pattern is the reverse to long-sighted learners – the combined inheritance mechanism outperforms recombination-only for all numbers of models.

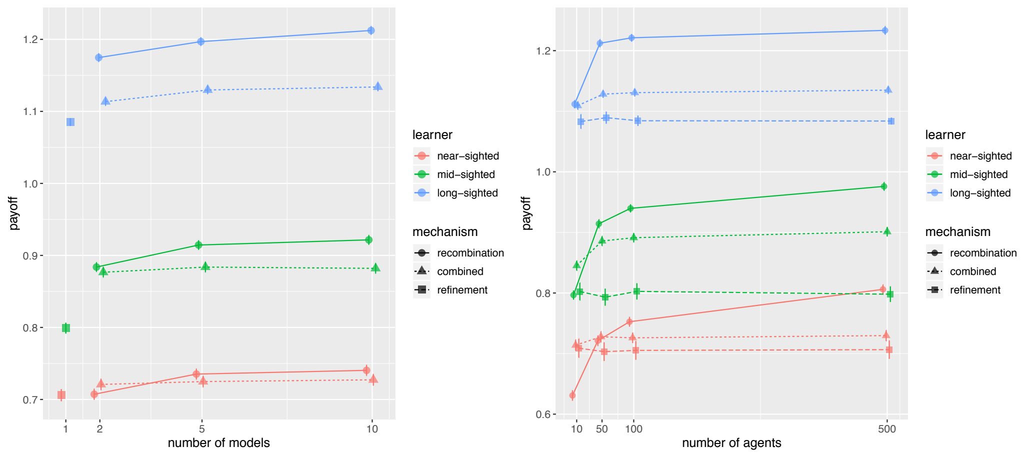

Figure S9 – the effect of (left) the number of models and (right) the number of agents: normalised payoff for each inheritance mechanism relative to the average payoff of 10,000 individual long-sighted learners, with the shape indicating the mechanism and the colour indicating the learning algorithm, across four values for the number of models (1, 2, 5, and 10). By definition, refinement alone involves purely copying from one model. The points plot the values in the final round (after 60 generations), averaged over 500 repeated simulations, averaged over all other parameter combinations (σ and n_a), with bars indicating two standard errors.

Similarly, the total number of agents in the population did not affect payoff considerably for either the combined mechanism, or the refinement alone mechanism (Fig. S6 right). For recombination only, however, an increase in the number of agents was associated with an increase in payoff, particularly for near-sighted learners, suggesting that the agents with the least knowledge about dependencies benefitted the most from the collective knowledge of the population. Therefore, learners using recombination show a clear increase in payoff with population size (and this increase is steady, Fig. S6), while learners using the combined mechanism, if anything, seem to decrease in payoff in a larger population. Again, there was an interaction between inheritance mechanism, learning algorithm, and the number of agents – agents using the recombination only mechanism performed consistently better than the agents using the other mechanisms when they were long-sighted learners, but near-sighted and mid-sighted learners in the recombination only mechanism only exceeded agents using the other inheritance mechanisms for large population sizes. When the population size was small, agents using the combined mechanism actually performed better.

9. Mutation

In addition to the stochasticity inherent to both recombination and refinement, we assumed learning is imperfect by implementing error as a mutation rate that determines how often agents mistakenly learn. In every round, for every agent, each step choice can be replaced with a different, randomly selected choice, with a probability equal to the mutation rate m. However, mutation rate only considerably affected the results when very high (Fig. S7). We see a considerable dip in payoffs for $m = 0.1$, which for a repertoire of 10 steps translates into an average of one mutated step every round for every agent. Interestingly, agents in the recombination only mechanism suffer much more because of mutation than agents in the other mechanisms, suggesting refinement provides a mechanism that mitigates errors to a certain extent.

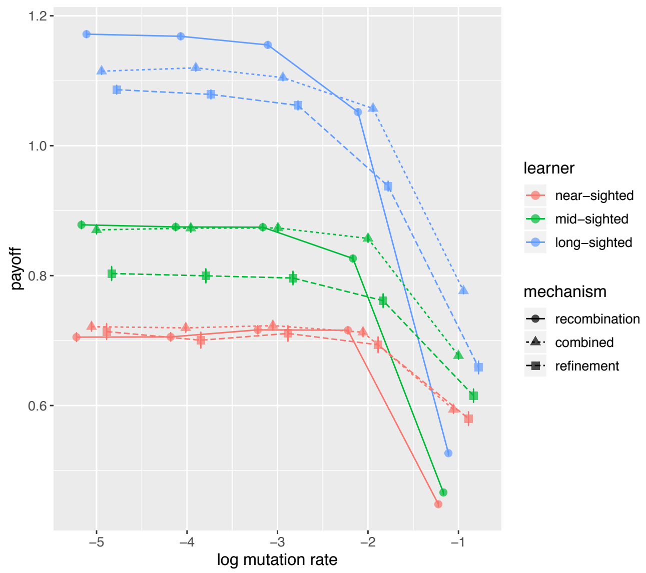

Figure S10 – the effect of mutation rate: normalised payoff for each inheritance mechanism relative to the average payoff of 10,000 long-sighted individual learners, where the shape indicates the mechanism and the colour indicates the learning algorithm, across five values of the mutation rate (m = [0, 0.0001, 0.001, 0.01, 0.1]), on the log10 scale for ease of visualisation. The points plot the values in the final round (after 30 generations), averaged over 200 repeated simulations, and averaged over all other parameter combinations (σ, n_a, and n_p), with bars indicating two standard errors.

10. Diversity

In addition to the diversity measure mentioned in the main manuscript, we explored three different measures of diversity, finding very similar patterns. The distance between two repertoires is measured as the average of the difference between choices at each step. For instance, the difference between repertoires r1 = [a,b,c] and r2 = [x,y,z] is $\frac{|a-x|+|b-y|+|c-z|}{3}$. Evenness (Pielou's evenness index) is a population-level measure that quantifies the flatness of a distribution. It is a measure used in quantifying species evenness in ecological communities based on the Shannon–Wiener diversity index, given by:

$$J = \frac{-\sum_{i-1}^{S} p_i \, ln \, p_i}{\ln(S)}$$

where S is the number of species present in a sample, and p_i is the relative frequency of species i in the sample. In our case, we are applying it step by step to measure the distribution of options, so for each step, S is the number of possible options and p_i is the number of repertoires that contain p_i for that step. This results in n_t values for J, which we averaged for the figure below. Finally, we measured how many distinct repertoires exist in the population at the end of the simulation.

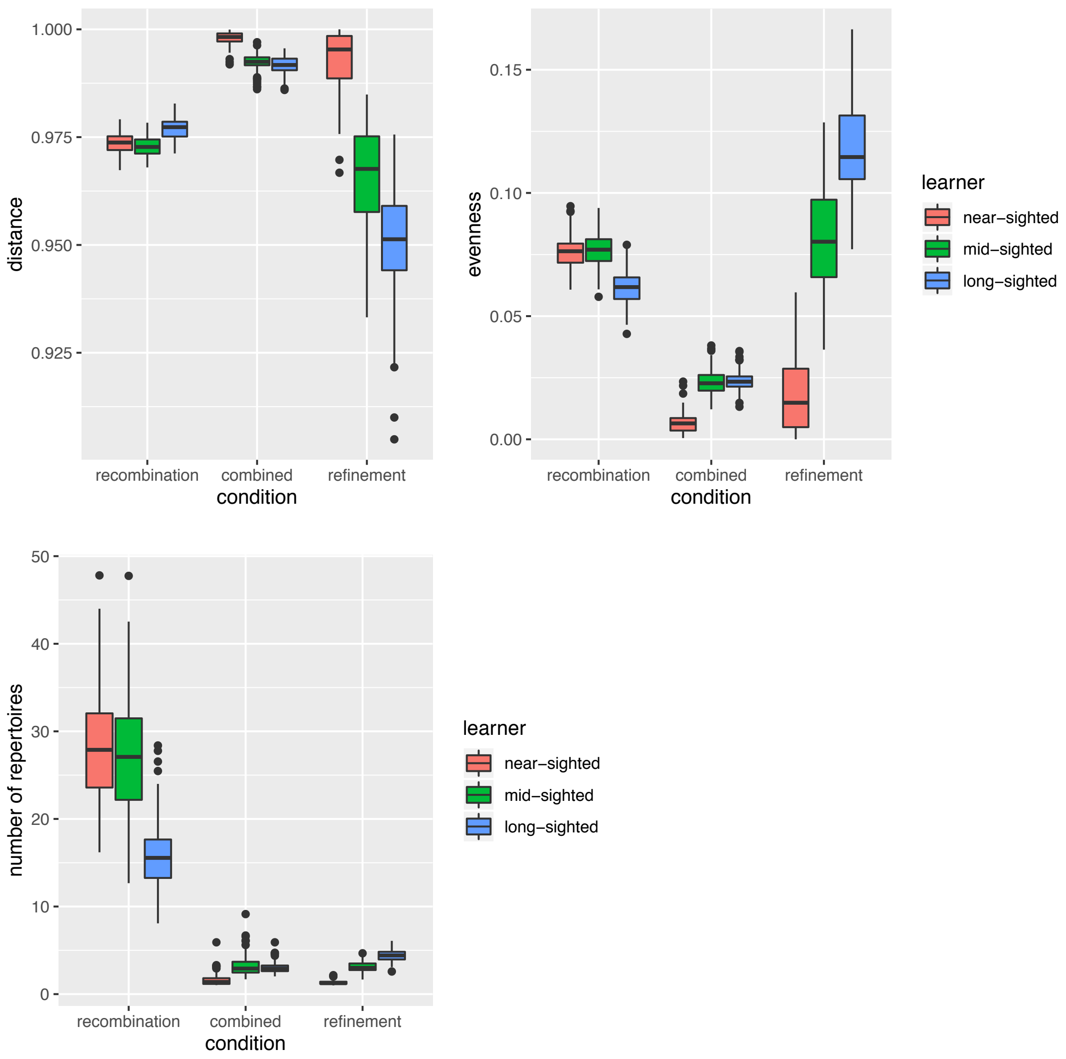

Figure S11 – distributions of repertoire distance, evenness, and number of repertoires at the end of 30 rounds, averaged over 100 simulation repeats and all parameter combinations for near-, mid-, and long-sighted learners, for the three inheritance mechanisms.

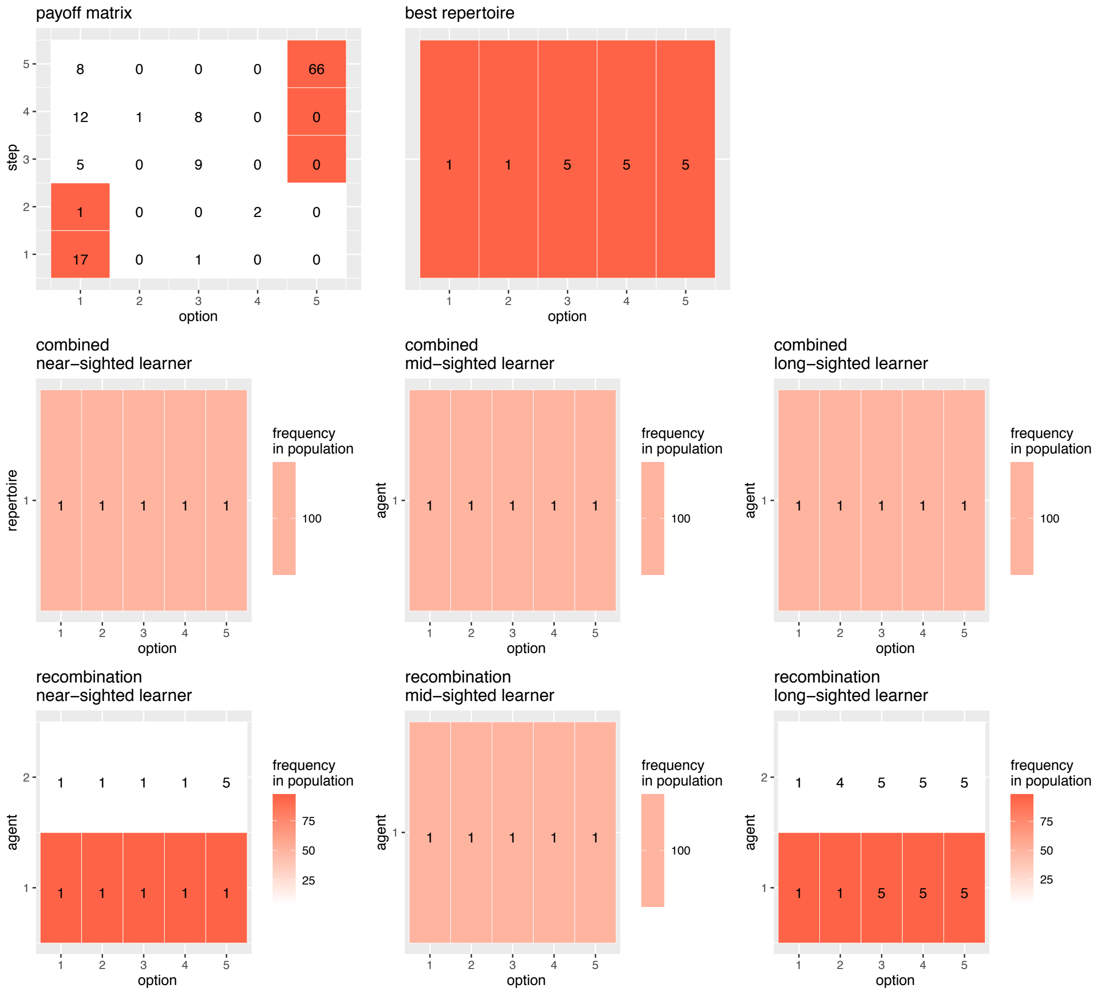

Figure S12 – One example run of 5 steps, each with 5 options, comparing a perfect (optimal) repertoire to the repertoires in the population after 30 runs for the combined and the recombination mechanism for 100 agents. Recombination maintains more variation in the population (here, near-sighted learners and long-sighted learners converge on two distinct repertoires, one more frequent than the other), while agents using the combined mechanism converge on a repertoire that is too uniform (i.e. the same option was chosen for all the steps)
